# Screening antivirals with a mCherry-expressing recombinant bovine respiratory syncytial virus: a proof of concept using cyclopamine

**DOI:** 10.1186/s13567-023-01165-x

**Published:** 2023-04-17

**Authors:** Jenna Fix, Delphyne Descamps, Marie Galloux, Cécile Ferret, Edwige Bouguyon, Siamak Zohari, Katarina Näslund, Sara Hägglund, Ralf Altmeyer, Jean-François Valarcher, Sabine Riffault, Jean-François Eléouët

**Affiliations:** 1 Université Paris-Saclay, INRAE, UVSQ, VIM, 78350 Jouy-en-Josas, France; 2 Department of Microbiology, National Veterinary Institute (SVA), Uppsala, Sweden; 3 Department of Clinical Sciences, Swedish University of Agricultural Sciences, Uppsala, Sweden; 4 Medusa Therapeutics Ltd, Hong Kong SAR, China

**Keywords:** Bovine respiratory syncytial virus, mCherry reverse genetics, antiviral drugs, cyclopamine

## Abstract

Bovine respiratory syncytial virus (BRSV) is a pathogenic pneumovirus and a major cause of acute respiratory infections in calves. Although different vaccines are available against BRSV, their efficiency remains limited, and no efficient and large-scale treatment exists. Here, we developed a new reverse genetics system for BRSV expressing the red fluorescent protein mCherry, based on a field strain isolated from a sick calf in Sweden. Although this recombinant fluorescent virus replicated slightly less efficiently compared to the wild type virus, both viruses were shown to be sensitive to the natural steroidal alkaloid cyclopamine, which was previously shown to inhibit human RSV replication. Our data thus point to the potential of this recombinant fluorescent BRSV as a powerful tool in preclinical drug discovery to enable high throughput compound screening.

## Introduction, methods and results

Bovine (BRSV) belongs to the *Orthopneumovirus* genus of the *Pneumoviridae* family [1]. It is a major cause of respiratory disease in young calves and is responsible for large economic losses worldwide in cattle [2]. More specifically, BRSV infection can provoke an acute lower respiratory disease in calves that shares many clinical and virological features compared to bronchiolitis symptoms induced by human RSV (HRSV) in infants [3]. The clinical picture is characterized by respiratory symptoms and the severity can range from mild to severe with sometimes even fatal outcome [4]. Control of BRSV infection is a priority in bovine production. Bacterial superinfections of BRSV-infected calves are frequent and contribute to the morbidity and mortality of infected animals. During epidemics, treatment of cattle with antibiotics is generally implemented to prevent bacterial superinfection [2]. However, this strategy is more and more questionable since it can increase the risk of emergence of antibiotic-resistant microbes, making BRSV an indirect threat to human health. Commercially available attenuated and killed BRSV vaccines are not satisfactory in terms of protection, and induce only a limited duration of immunity [5, 6]. Like for HRSV, a stabilized recombinant prefusion BRSV F protein was recently shown to be protective against a BRSV challenge in calves [7, 8]. Nevertheless, even if BRSV vaccines have shown efficacy that lasts at least three months, outbreaks are still very common and effective treatments limiting virus replication and its consequences are needed to limit the morbidity and mortality caused by this virus. Affordable drugs would reduce the economic impact of BRSV and improve animal welfare.

Recombinant viruses expressing reporter genes have shown their value in facilitating in vitro and in vivo experiments, including the study of vaccine efficacy and the search of antiviral compounds. Here, to facilitate the study of BRSV replication in cellula and the screening of antivirals, we developed a new reverse genetics system for BRSV derived from a field strain isolated in Sweden and expressing a mCherry reporter gene.

### Isolation and sequencing of a BRSV field strain

The present work was based on the BRSV/Sweden/HPIG-SLU-620-Lövsta/2016 strain (BRSV Lövsta). This strain was isolated in January 2016 during a major BRSV outbreak in Sweden, leading to virus spreading within the research dairy herd of the Swedish University of Agricultural Science, Uppsala. The severity of the BRSV outbreak caused by this strain can be considered as moderate, based on clinical observations and economic impact [9]. Briefly, several animals developed fever (up to 41.1 °C) and inappetence. Nasal secretions were collected with swabs (UTM™virus swab, Copan, Italy) from several animals with acute respiratory signs, and among these a 3 months old dairy heifer, with tachypnoea, serous nasal discharge and a rectal temperature of 40.2 °C. The nasal swab samples of this white dairy heifer were frozen at −80 °C immediately after collection, for BRSV isolation on bovine nasal turbinate cells, as previously described [10]. A cytopathogenic effect was observed during the first isolation and after one passage, four days post-inoculation. The cell culture was then frozen at −76 °C until RNA extraction and sequencing. Nucleic acids were extracted using Viral NA Magnetic Beads kits in an ArrowTM 2 extraction robot (DiaSorin, Saluggia, Italy). For the extraction, 250 µL of the cell supernatant was digested with 10 µL ≥ 800 U/mL proteinase K (Sigma-Aldrich, Saint Louis, MO, USA), and run in the extraction robot according to the manufacturer’s recommendations. According to the manufacturer’s instructions, library construction was performed using NEXTERA-XT kit (Illumina Inc. San Diego, CA, USA). The Agilent 2100 Bioanalyzer (Agilent Technologies. Santa Clara, CA, USA) was used to assess the quality of the obtained libraries. The libraries were sequenced on a MiSeq Instrument (Illumina Inc. San Diego, CA, USA) available at the Department of Microbiology, National Veterinary Institute, Uppsala, Sweden, using a Miseq Reagent Kit v2 in a 600-cycle paired-end run. Quality analysis, filtering and de novo assembly of the raw reads were performed by CLC genomics workbench 10.0.1 (CLC bio, Aarhus, Denmark). After quality assessment and trimming, a total of 638 916 cleaned reads were obtained with an average length of 200 base pairs (bp). The trimmed reads were assembled into 4200 contigs with an average length of 1350 bp. The full genome cDNA for the strain BRSV/Sweden/HPIG-SLU-620-Lövsta/2016 (BRSV Lövsta) was found to be 15 140 bp in length with a 33.83% G + C content. Genome annotation was done by using CLC genomics workbench 10.0.1. The genome contains 11 genes and has a genome sequence and genome organization similar to the reference BRSV strain ATCC51908 (GenBank accession number NC_038272). The complete genome sequence of the Swedish BRSV strain (Sweden_HPIG-SLU-620-Lövsta_2016) was submitted to the GenBank database under accession number MG947594.

### Design and recovery of an infectious recombinant BRSV-mCherry from cDNA

**Figure 1 Generation of recombinant BRSV.**
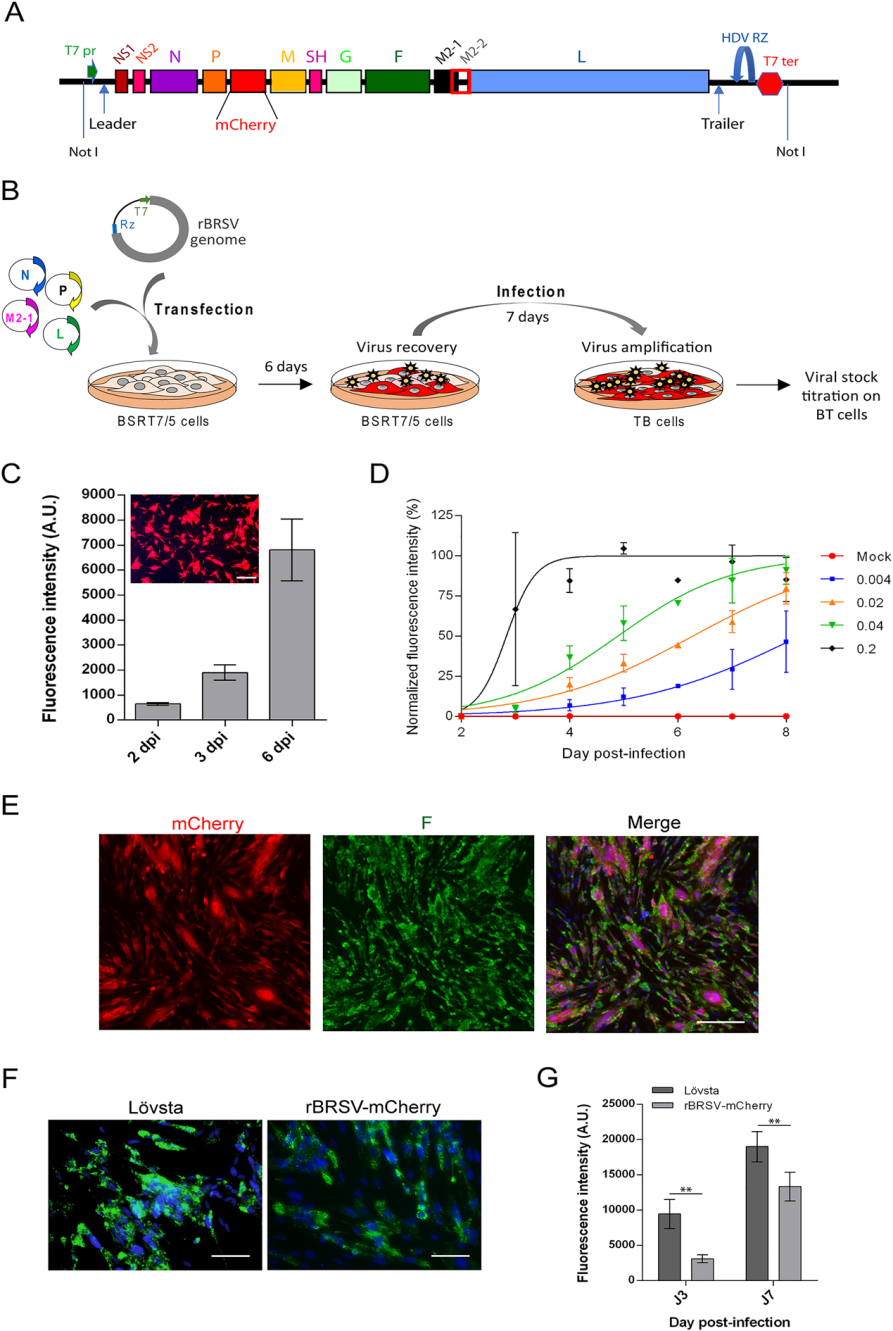
**A** Diagram of the rBRSV-mCherry cDNA construct cloned in the pACNR1180 low copy vector at NotI site. The synthetic cassette made by DNA synthesis contains the full-length BRSV Lövsta cDNA, a T7 promoter (T7 pr) fused to the Leader region, an HDV ribozyme (RZ) at the 3’ end of the antigenome, followed by a T7 transcription terminator (T7 ter). The expressed antigenomic RNA contains 3 additional guanosines at the 5’ end, an exact 3’ end (Trailer) sequence, and an additional transcription unit to express mCherry in infected cells. **B** Schematic representation of the protocol of recovery and amplification of rBRSV-mCherry. **C** Quantification of rBRSV-mCherry replication at 2, 3, and 6 dpi by fluorescence measurement using a Tecan infinite M200Pro spectrofluorometer. BT cells were infected with an undiluted viral stock with a MOI of 0.2 and data are representative of one experiment performed in quadruplicates. Standard deviations are indicated. A representative image of BT cells infected with rBRSV-mCherry at 6 dpi is shown. Cell nuclei were stained with Hoechst 33342. Scale bar, 100 μm. **D** Quantification of rBRSV-mCherry replication at different MOI from 2 to 8 dpi by fluorescence measurement using a Tecan infinite M200Pro spectrofluorometer. Data were normalized based on the maximal fluorescence intensity obtained and by the value of fluorescence of untreated infected cells. Data are representative of two experiments made in duplicates, except data at 6 dpi which correspond to simplicates. Standard deviations are indicated. **E** Images of BT cells infected with rBRSV-mCherry (MOI 0.04) at 8 dpi. Cells were fixed and labelled with anti-F antibody (green). Cell nuclei were stained with Hoechst 33342. Scale bar, 100 μm. **F, G** Comparison of Lövsta and rBRSV-mCherry replication at 3 and 7 dpi. Cells were infected at MOI 0.02, fixed at 3 or 7 dpi and labelled with anti-F antibody, and nuclei stained with Hoechst 33342. Scale bar, 100 μm **F** Fluorescence was quantified using a Tecan infinite M200Pro spectrofluorometer. Data were normalized by the value of fluorescence of untreated infected cells and are representative of one experiment performed with 6 replicates. Data are means ± SD, ** *p* < 0.05.

An expression cassette was designed for the expression of the antigenomic RNA of the field strain isolate Sweden_HPIG-SLU-620-Lövsta_2016 with an additional transcription unit coding for the red fluorescent mCherry protein (Genbank accession numbers ON110491). The additional mCherry gene was placed between P and M genes with gene start and gene end sequences from the N gene upstream and downstream the mCherry gene. A T7 RNA polymerase promoter was placed upstream the Leader region such that antigenomic RNA would be transcribed with three supplementary G residues at the 5’ end. The hepatitis delta virus (HDV) antigenome ribozyme sequence was fused to the last nucleotide of the trailer sequence to achieve correct cleavage at the 3’ end (Figure 1A). This cassette was made by DNA synthesis by GenScript, Leiden, Netherlands, and cloned at a unique NotI restriction site in the low-copy vector pACNR-1180 [11] and entirely controlled by sequencing.

The BHK-21-derived BSRT7/5 clone constitutively expressing the phage T7 RNA polymerase was originally made to facilitate reverse genetics of BRSV, BRSV growing much more slowly than HRSV in cultured cells [12]. It was also previously shown that cross-recognition of BRSV cis-acting elements by HRSV allows the rescue of BRSV from cDNA using HRSV N, P, L, M2-1 genes [13, 14]. BSRT7/5 cells and bovine turbinate cells (BT cells, ATCC number CLR-1390) were grown in Dulbecco Modified Essential Medium (Lonza) supplemented with 10% fetal calf serum (FCS) or horse serum, respectively, with 2 mM glutamine and 1% penicillin-streptomycin. For recombinant BRSV (rBRSV) recovery, BSRT7/5 cells were grown in 32-mm-diameter dishes (6-well plates) to 70–80% of confluence. One hour before transfection, cells were washed once with 1 mL of Opti-MEM medium (Gibco). Cells were then transfected with a plasmid mixture containing 1.25 µg of full-length plasmid prBRSV-mCherry, 1 µg of pN, 1 µg of pP, 0.25 µg of pM2-1, and 0.5 µg of pL expression vectors for P, N, L and M2-1 genes from HRSV long strain under the control of a T7 promoter [15] (Figure 1B). Transfection experiments were carried out with Lipofectamine 2000 (Invitrogen). The transfection medium was removed 24 h post-transfection; cells were washed and maintained in Dulbecco’s modified Eagle Medium containing 2% horse serum. Visualization of cells expressing mCherry indicated the capacity to recover the recombinant virus. Six days post-transfection, cells were scraped, vortexed, and cellular debris were removed by pelleting at 800 × *g*. The undiluted supernatants were then used to amplify the virus on BT cells during 7 days in DMEM containing 2% horse serum with 2 mM glutamine, and 1% penicillin-streptomycin. Red fluorescence was quantified at 2, 3, and 6 days post-infection (dpi) by using a Tecan infinite M200Pro spectrofluorometer, allowing to validate the efficient propagation of the virus on BT cells (Figure 1C). After two rounds of amplification on BT cells, the virus titre was determined using standard protocol. Briefly, BT cells were infected with serial dilutions of virus during 6 days, fixed with 4% paraformaldehyde in PBS for 20 min at room temperature, rinsed with PBS, and permeabilized with 1% BSA, 0.1% Triton X-100 in PBS for 10 min. Cells were incubated for 1 h with mouse anti-F antibody (Serotec) [16] in PBS-1% BSA, rinsed with PBS, and incubated with anti-mouse Alexa Fluor 488-conjugated secondary antibody (Invitrogen). The infected cells were then counted with a Nikon TE200 microscope equipped with a CoolSNAP ES2 (Photometrics) camera. Images were processed with Meta-Vue software (Molecular Devices). The titres of Lövsta and rBRSV-mCherry viruses used in the present study were 5.5 × 10^4^ pfu/mL and 1 × 10^4^ pfu/mL, respectively.

We then assessed the capacity of this recombinant virus to replicate on BT cells. Given the low titre of the viral stock, cells were infected at MOI 0.2 (undiluted inoculum), 0.04, 0.02 or 0.004. The rBRSV-mCherry fluorescence was quantified daily from 2 to 8 dpi to determine the kinetics of replication (Figure 1D). Fluorescence values were normalized based on the maximal fluorescence intensity, which was reached as soon as 4 days post-infection for cells infected at MOI 0.2, and 8 dpi for cells infected at MOI 0.04. Of note, this intensity of fluorescence corresponds to nearly full infection of the cell culture, as assessed by the observation by epifluorescence of cells infected with MOI 0.04 at 8 dpi (Figure 1E, left panel). Our results revealed that the kinetics of replication were proportional to the initial viral load and highlighted the slow kinetics of replication of rBSRV for lower MOI. Important standard deviations were observed for the highest MOI (0.2) tested. We hypothesise that these variations could be due to the strong tendency of BRSV to form sticky filaments, which induced bias in the infection at this virus concentration. Finally, cells infected at MOI 0.04 fixed at 8 dpi were labelled with anti-F antibody, and nuclei stained for observation by fluorescence microscopy. As shown on Figure 1E, the cells expressing F also expressed mCherry, thus validating the stability of mCherry expression. It is noteworthy that mCherry fluorescence allowed to clearly identify infected cells compared to F labelling, commonly used for BRSV titration, but which displayed diffuse fluorescence.

We then compared the replication of rBRSV-mCherry and the Lövsta field strain on BT cells. Based on the kinetics of replication of rBRSV-mCherry and due to the low virus titres, cells were infected at MOI 0.02. At 3 and 7 dpi, cells were fixed and labelled with anti-F antibody to detect infected cells, before quantification of replication by fluorescence measurement (Figures 1F and G). Our results revealed that the field strain Lövsta replicated more efficiently than rBRSV-mCherry.

Altogether, our data confirmed that rBRVS-mCherry replicates efficiently and that mCherry fluorescence measurement, which allows to follow the infection each day on live cells, can facilitate BRSV replication quantification. This virus could thus represent a new tool to screen antivirals.

### Antiviral potency of cyclopamine against recombinant BRSV-mCherry

**Figure 2 Inhibition of BSRV replication by cyclopamine in cell culture.**
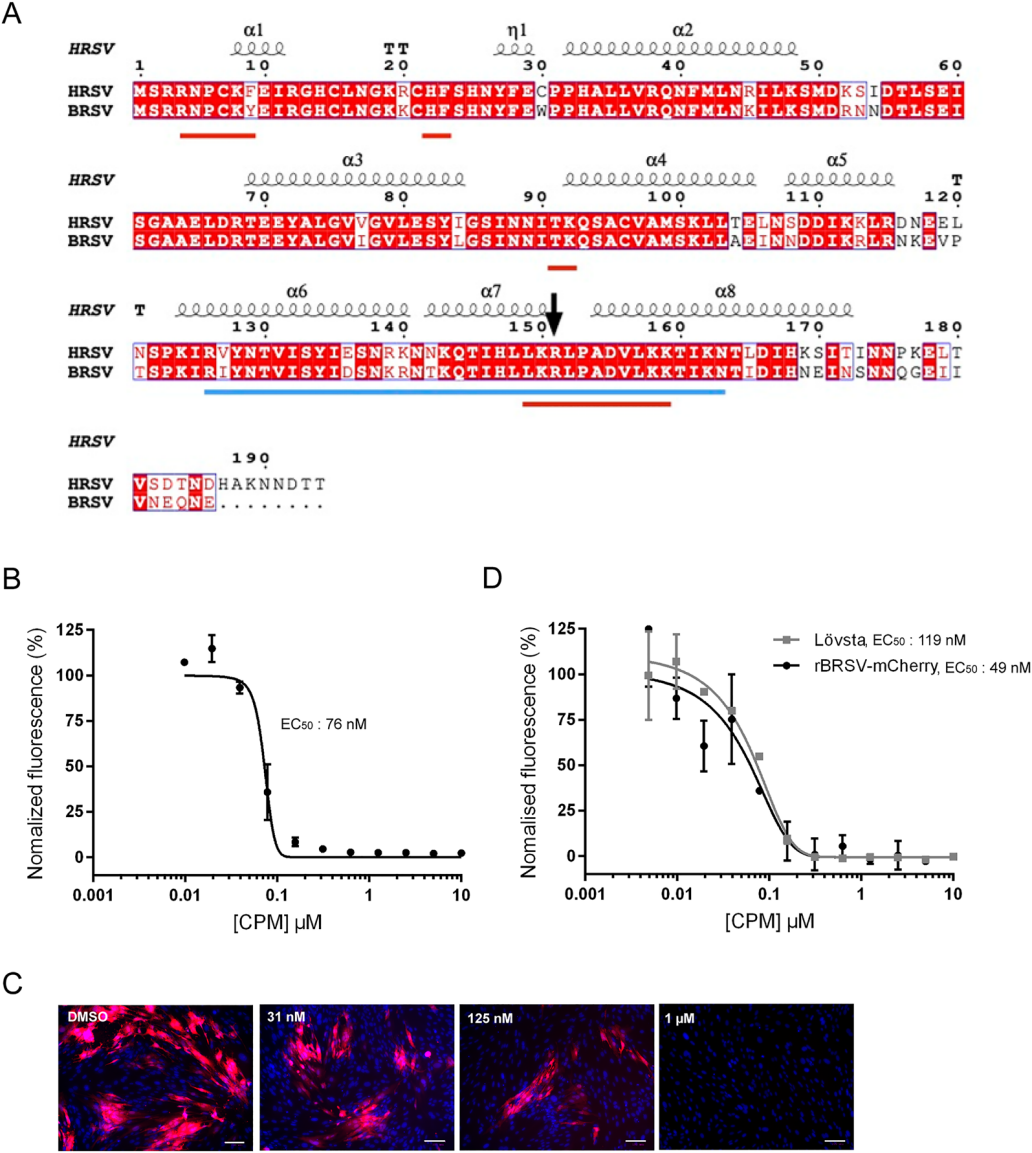
**A** Amino acid sequence alignment of M2-1 of HRSV strain Long and BRSV Lövsta strain (GenBank accession codes AMA66581.1 and QBK50987.1, respectively) by ClustalW and prepared with ESPript3. The R151 residue is indicated by an arrow. RNA and P-binding domains previously identified [20, 23], are underlined by red and blue lines, respectively. **B** BT cells in 96-well plates were infected with rBRSV-mCherry at MOI 0.02 and incubated in the presence of serial dilutions of CPM (initial concentration of 10 µM, serial dilutions at ½). The mCherry fluorescence was quantified on live cells at 7 dpi using a Tecan infinite M200Pro spectrofluorometer. Data were normalized by the value of fluorescence of untreated infected cells. Data are representative of three experiments made in duplicates. Standard deviations are indicated. **C** Images of BT cells infected with rBRSV-mCherry and treated with CPM at different concentrations at 7 dpi. Cell nuclei were stained with Hoechst 33342. Scale bar, 100 μm. **D** BT cells in 96-well plates were infected with either Lövsta or rBRSV-mCherry at MOI 0.02 and incubated in the presence of serial dilutions of CPM (initial concentration of 10 µM, serial dilutions at ½). Cells were fixed at 7 dpi, immunolabelled with anti-F antibody, followed by incubation with secondary antibody coupled to Alexa488. Fluorescence intensity was measured using a Tecan infinite M200Pro spectrofluorometer. Data were normalized by the value of fluorescence of corresponding untreated infected cells. Data are representative of two experiments made in duplicates. Standard deviations are indicated.

We have previously found that the natural steroidal alkaloid cyclopamine (CPM) extracted from *Veratrum californicum* can inhibit HRSV replication in cell culture and in vivo in mouse [17, 18]. We have also shown that a single R151K mutation in the viral M2-1 protein was sufficient to confer resistance to CPM, indicating that the essential M2-1 transcription factor is playing a critical role in the mechanism of action of CPM. Alignment of BRSV and HRSV M2-1 sequences shows that M2-1 proteins are highly conserved between BRSV and HRSV, excepted in the C-terminal disordered region (Figure 2A). In particular, R151 and all surrounding residues were conserved, suggesting that CPM could also be active against BRSV. Of note, the R151 residue is located on the surface of the M2-1 core region, in a positively charged region made by helices α6, α7, and α8 that binds to P and RNA in a competitive manner [19, 20]. We therefore tested CPM antiviral activity against BRSV on BT cells infected with rBRSV-mCherry at MOI = 0.02. Two hours post-infection, the medium was changed for medium containing serial dilutions of CPM (with an initial concentration of 10 µM in DMSO and ½ serial dilutions). Quantification of viral replication was performed by fluorescence measurement on live cells at 7 dpi using the Tecan spectrofluorometer. Values of nontreated infected and noninfected BT cells were used for standardization and normalization. The half maximal effective concentration (EC_50_) was determined by fitting the data to the dose-response curve implemented in GraphPad version 8 software. Cell survival was quantified using the CellTiter-Glo Luminescent cell viability assay (Promega). As previously determined on human cells [17], CPM was not toxic for BT cells up to 10 µM concentration (not shown). As shown in Figure 2B, CPM inhibited rBRSV-mCherry replication in a dose-dependent manner, with a calculated EC_50_ of 76 nM. These data were confirmed by observation of mCherry fluorescence and nuclei stained with Hoechst 33342 on fixed rBRSV-mCherry-infected cells (Figure 2C). To confirm these results, similar experiment was performed by infecting BT cells with either the field strain Lövsta or rBRSV-mCherry. At 7 dpi, cells were fixed and labelled with anti-F antibody and secondary antibody coupled to Alexa 488, before fluorescence quantification. Given the difference in viruses replication efficiency, data were normalised for each virus by using the corresponding untreated condition. As shown on Figure 2D, CPM treatment efficiently impaired the replication of both viruses, with calculated EC_50_ of 119 nM and 49 nM for Lövsta and rBRSV-mCherry, respectively. The slight difference between EC_50_ measured by either mCherry or F fluorescence (76nM versus 49 nM) could be due to the treatments of cells for F immunofluorescence compared to the epifluorescence of mCherry on live cells. But these results correlate with those previously obtained for HRSV in similar experimental conditions, with an EC_50_ close to 126 nM [17].

Altogether, these data show that CPM displays similar antiviral activity against HRSV and BRSV and validate the use of rBRSV-mCherry for screening antivirals.

## Discussion

The aim of this work was to set up a new reverse genetics system that could facilitate the study of BRSV replication and the screening of antiviral compounds. We show that the BRSV Lövsta-derived rBRSV-mCherry is a valuable tool since infected cells can be easily monitored for at least one week without cell fixation. Although many reverse genetics and minigenome systems were developed and widely used in HRSV research, drug and vaccine discovery studies, their use has been more limited for BRSV [12, 14, 21, 22]. To our knowledge, all these publications are based on two reverse genetics systems that were both derived from the same BRSV ATue51908 strain (ATCC51908) [12, 14]. Our new reverse genetics system is based on a recent field-isolate and represents a new tool enabling medium and high throughput screening of RSV antiviral candidates in cell cultures. However, in our hands the viral titres of both rBRSV-mCherry and Lövsta were low (5.5 × 10^4^ pfu/mL and 1 × 10^4^ pfu/mL respectively), limiting the maximum MOI to 0.2 pfu/mL; this issue could limit their use for high throughput screenings and studies with antiviral compounds. Optimization of virus stocks to improve MOI will thus be required. Although the insertion of an additional gene in the viral genome allowed to recover a recombinant virus which replicates efficiently, its replication was slower compared to the wild type virus. However, the comparison is questionable as it is likely that Lövsta viral stock corresponds to a population of viral genomes (quasispecies), whereas rBRSV-mCherry is derived from a unique sequence of viral genome.

As a proof of concept, we used the previously identified CPM compound that was shown to inhibit HRSV replication both in cell culture and in mouse models. Although the molecular target of CPM has not been reported yet, R151K substitution in M2-1 protein was associated with viral resistance against CPM, indicating that M2-1 participates in the sensitivity to CPM. Because of the high homology between BRSV and HRSV M2-1, we reasoned that CPM would also inhibit BRSV replication. We used our novel reverse genetics system to show that CPM inhibited BRSV and HRSV with similar potency, paving the way for the development of CPM analogues for the treatment of HRSV or BRSV infections. Inhibition of rBRSV-mCherry in cell culture has predictive value for the inhibition of BRSV in calves. In turn, BRSV may serve as a natural animal model that shows all the hallmarks of human RSV infection, notably lung replication, pathology and disease. Given the high amino acid homology between human and bovine RSV replication machinery proteins, the predictive value of the BRSV calf model for HRSV inhibition in humans should be further explored. The rBRSV-mCherry virus combined with the BRSV calf model is a valuable tool for replication-targeted antivirals, providing animal proof of concept and de-risking prior enabling human clinical trials.
